# Acupuncture plus wet cupping therapy for post stroke depression: a randomized controlled trial

**DOI:** 10.3389/fpsyt.2026.1779004

**Published:** 2026-04-27

**Authors:** Dong Li, Hang Gao, Jin Li, Larissa Tao, Lili Zhu, Weidong Shen, Wa Cai

**Affiliations:** 1 Department of Acupuncture, The Affiliated People’s Hospital of Fujian University of Traditional Chinese Medicine, Fuzhou, China; 2 Department of Acupuncture, Shanghai Shuguang Hospital Affiliated to Shanghai University of Traditional Chinese Medicine, Shanghai, China; 3 Department of Rehabilitation, Shanghai Shuguang Hospital Affiliated to Shanghai University of Traditional Chinese Medicine, Shanghai, China

**Keywords:** acupuncture, hypothalamic-pituitary-adrenal axis, inflammatory factors, post-stroke depression, wet cupping therapy

## Abstract

**Objective:**

To explore the preliminary clinical efficacy and safety of acupuncture plus wet cupping on post-stroke depression (PSD) and to investigate its potential associations with changes in the hypothalamic-pituitary-adrenal (HPA) axis and inflammatory factors.

**Methods:**

130 mild-to-moderate PSD patients were randomized into two groups: an acupuncture plus wet cupping therapy group (AC group, n=65) and an acupuncture-only group (Acu group, n=65). The primary outcome was the change from baseline (pre-treatment) in the Hamilton Depression Scale-24 (HAMD-24) score at the end of week 4 (post-treatment). Secondary outcomes included the change from baseline in the HAMD-24 score at the 8 weeks after the 4-week treatment period (follow-up), the changes from baseline (pre-treatment) in the Self-rating Depression Scale (SDS), National Institute of Health Stroke Scale (NIHSS), and Montgomery-Asberg Depression Rating Scale (MADRS) at the end of week 4 (post-treatment) and 8 weeks after the 4-week treatment period (follow-up). Hematological indicator outcomes were measured at baseline and the end of week 4 (post-treatment), including cortisol (Cort), adrenocorticotropic hormone (ACTH), interleukin (IL-1β, IL-2, IL-6, IL-8, IL-10, IL-17), and tumor necrosis factor-α (TNF-α).

**Results:**

118 patients completed the trial. AC group showed greater reductions in HAMD-24, SDS, NIHSS, MADRS scores post-treatment and follow-up (all P<0.01), and more significant regulation of Cort, ACTH, pro-inflammatory cytokines (IL-1β, etc.) and anti-inflammatory IL-10 (all P<0.01). No serious adverse events occurred.

**Conclusion:**

In this randomized controlled trial, acupuncture plus wet cupping might show comparative superiority over acupuncture monotherapy in improving PSD symptoms and neurological function, and was accompanied by favorable changes in HPA axis and inflammatory factors. The findings reflect incremental therapeutic benefits rather than definitive efficacy beyond placebo or standard care for PSD.

**Clinical trial registration:**

## 1 Introduction

Stroke is a leading cause of disability and mortality worldwide, particularly among middle-aged and elderly individuals. Post stroke depression (PSD) is the most common neuropsychiatric complication following stroke, affecting approximately 18-33% of patients (1–3). PSD not only hinders rehabilitation but also worsens functional impairments and reduces quality of life. Patients commonly present with low mood, anhedonia, sleep disturbances, poor appetite, and cognitive difficulties; severe cases may involve hallucinations or suicidal ideation (4).

Conventional treatment options for PSD include psychotherapy, pharmacotherapy, and physical therapies. Pharmacotherapy, the most widely used first-line intervention in clinical practice, primarily involves selective serotonin reuptake inhibitors (SSRIs, e.g., fluoxetine, sertraline), serotonin-norepinephrine reuptake inhibitors (SNRIs, e.g., venlafaxine), and tricyclic antidepressants (TCAs) (5). While effective, these approaches may carry side effects or limitations, reducing patient compliance (5–7). This highlights the urgent need for alternative interventions that are both effective and well-tolerated.

In recent years, acupuncture has gained increasing importance in the management of PSD, earning recognition from both patients and their families, and drawing growing attention from the research community. Previous clinical trials conducted by our team have demonstrated that acupuncture can effectively alleviate depressive symptoms in PSD and provides unique advantages in improving patients’ quality of life (8). Wet cupping therapy, which involves tapping the skin with a plum-blossom needle to induce mild bleeding followed by cupping, promotes the removal of stagnant blood and viscous secretions. This process is traditionally believed to eliminate blood stasis, generate new blood, and unblock meridians, which can enhance immune surveillance and nervous system function to generate a cascade of physiological responses (9). A cross-sectional study found that wet cupping and acupuncture can be used as complementary and alternative therapies to relieve depression (10).

No prior randomized controlled trial (RCT) has compared acupuncture monotherapy with acupuncture plus wet cupping in PSD while simultaneously assessing hypothalamic-pituitary-adrenal (HPA) axis and cytokine biomarkers, leaving a critical evidence gap for mechanism-driven integrative interventions. The pathogenesis of PSD is thought to involve an abnormal inflammatory response and dysregulation of the neuroendocrine system, particularly the HPA axis (11). Pro-inflammatory mediators have been identified as potential triggers of PSD, as they impair neurotransmitter homeostasis and downregulate the synthesis of brain-derived neurotrophic factor (BDNF) (12). With disease progression, these pathological changes can further activate inflammatory pathways, leading to glucocorticoid receptor (GR) dysfunction and hypersecretion of adrenocorticotropic hormone (ACTH). This process disrupts HPA axis homeostasis and results in pathological increases in cortisol (Cort) levels (13, 14). Concurrently, heightened oxidative stress weakens the antioxidant defense system, exemplified by superoxide dismutase (SOD) inhibition. Mitochondrial dysfunction and the accumulation of oxidative metabolites further aggravate HPA axis dysregulation through aberrant activation of the GR-ACTH signaling network (15). This vicious cycle of neuroendocrine imbalance, oxidative stress, and inflammation ultimately drives the progression of PSD.

Mechanistically, acupuncture is hypothesized to regulate HPA axis function by modulating central neurotransmitter systems and inhibiting excessive ACTH/Cort secretion, while wet cupping may enhance immunoregulation by reducing pro-inflammatory cytokine release and promoting blood circulation to resolve “stasis”, which is a key TCM pathogen linked to inflammation and neuroendocrine dysfunction (16, 17). Their combination may exert synergistic effects by simultaneously targeting both HPA axis dysregulation and inflammatory responses, addressing the multi-pathway pathogenesis of PSD. We focused on Cort, ACTH, interleukin (IL), and tumor necrosis factor-α (TNF-α), given their central role in the neuroendocrine-immune crosstalk underlying PSD. Cort and ACTH are direct biomarkers of HPA axis hyperactivity, while pro-inflammatory cytokines (IL-1β, IL-2, IL-6, IL-8, IL-17, TNF-α) and the anti-inflammatory cytokine IL-10 critically regulate neuroinflammation and synaptic plasticity, which are two pathophysiological processes closely linked to depressive symptom severity and neurological recovery after stroke (18–20). Given that PSD treatments often have adverse effects or limited adherence, low-risk alternatives such as acupuncture plus wet cupping deserve rigorous investigation to provide safer and more acceptable options for patients.

Therefore, this study was designed as an exploratory randomized trial to assess the preliminary efficacy and safety of acupuncture plus wet cupping for PSD, and to explore whether clinical improvements were accompanied by changes in Cort, ACTH, and inflammatory factors.

## 2 Materials and methods

### 2.1 Trial design

This is a single-center, parallel-group, randomized controlled trial comparing the efficacy of acupuncture alone with that of acupuncture plus wet cupping in patients with post-stroke depression. The study protocol and informed consent forms were approved by the Ethics Committee of Shanghai Shuguang Hospital Affiliated to Shanghai University of Traditional Chinese Medicine (Approval No: 2024-1442-025-01). The trial was conducted between May to December 2024, with participants recruited from the hospital’s Acupuncture and Rehabilitation Departments and social channels. Follow-up assessments were conducted at 8 weeks after completion of treatment. For clarity, “acupuncture-only group” is hereinafter referred to as the “Acupuncture group” (abbreviated as “Acu group”), and “acupuncture plus wet cupping therapy group” abbreviated as “AC group” throughout the manuscript.

### 2.2 Participants

#### 2.2.1 Diagnostic criteria

The diagnostic criteria were formulated with reference to “Diagnostic criteria of cerebrovascular diseases in China (version 2019)” (21) and “Chinese Expert Consensus of Post-stroke Depression and Clinical Practice Neuropsychology and Mood Disorder Committee of Society of Neurology Physician” (22).

##### 2.2.1.1 Stroke diagnostic criteria

1. Cerebral infarction: Acute onset of focal neurological deficits (rarely generalized dysfunction); Neuroimaging evidence (CT/MRI) of a responsible infarct lesion, or persistence of clinical symptoms for ≥24 hours; Exclusion of non-ischemic etiologies.
2. Intracerebral hemorrhage: Sudden onset of focal neurological deficits, with or without accompanying headache, vomiting, or altered consciousness; Cranial CT/MRI demonstrating an intracerebral hemorrhage lesion; Exclusion of traumatic or other secondary hemorrhagic causes.

##### 2.2.1.2 PSD diagnostic criteria

1. Core symptoms: At least 3 of the following 9 symptoms (must include either item 1 or 2) persisting for ≥1 week: Persistent low mood (subjectively reported or objectively observed); Marked reduction in interest or anhedonia; Persistent fatigue or decreased energy unrelated to physical exertion; Psychomotor retardation or agitation; Excessive guilt, pathological self-reproach, or feelings of worthlessness; Cognitive impairments (e.g., reduced decision-making ability, slowed thinking, or attention disturbances); Recurrent suicidal ideation or behaviors; Sleep rhythm disturbances (e.g., insomnia, early awakening, or hypersomnia); Appetite disorders with significant weight fluctuation.
2. Functional impairment: Symptoms resulting in at least one of the following: Significant clinical distress; Impairment in social, occupational, or other important areas of functioning.
3. Temporal association: Definite history of stroke; Depressive symptoms occurring within 1 year after stroke; Symptoms not exclusively present during an episode of delirium.

#### 2.2.2 Inclusion criteria

1. Meet the above diagnostic criteria.
2. the Hamilton Depression Scale-24 (HAMD-24) scores: 8–35 points, belonging to mild or moderate depression.
3. Age: 40–85 years old.
4. Gender: not limited.
5. Not receiving other antidepressant treatments.
6. Capable of providing informed consent, with no communication barriers and able to cooperate with treatments and assessments.

#### 2.2.3 Exclusion criteria

1. Consciousness-impaired patients.
2. Those with advanced cortical dysfunctions (agnosia/apraxia/aphasia).
3. Patients with severe cognitive impairment.
4. Those having progressive major organ diseases or multiple-system co-existing diseases.
5. Patients with coagulation disorders.
6. Those with a personal and/or family history of mental illness.
7. Alcoholics or psychotropic-drug-dependent individuals.
8. Patients who are afraid of needles or blood, or are uncooperative with treatment.
9. Patients unable to sign the informed consent form.

#### 2.2.4 Interventions

##### 2.2.4.1 Basic treatment

All participants received standard post-stroke management in accordance with clinical guidelines, including blood pressure control, glycemic regulation, lipid-lowering therapy, complication prevention, and rehabilitation measures (23).

##### 2.2.4.2 Acu group

1. Acupoint selection referring to clinical guideline (24), included Baihui (GV20), Sishencong (EX-HN1), Shenting (GV24), Taichong (LR3, bilateral), and Sanyinjiao (SP6, bilateral). Baihui (GV20) and Shenting (GV24) could calm the mind and regulate brain function, with Shenting (GV24) additionally stabilizing the will and inducing resuscitation. Sishencong (EX-HN1) could improve cognitive function and relieves emotional distress. Taichong (LR3) and Sanyinjiao (SP6) could soothe the liver, nourish blood, and regulate qi, while Taichong (LR3) specifically alleviates liver qi stagnation to target depressive symptoms (25–28).
2. Acupuncture manipulation: Sterile disposable acupuncture needles (0.25 mm × 40 mm, Wuxi Jiajian Medical Instrument Co., Ltd., China) were used for all interventions, which was performed by a licensed acupuncturist with at least 5 years of clinical experience. The acupuncturist underwent standardized training prior to the study initiation. Patients were placed in the supine position. After routine disinfection with 75% ethanol, sterile disposable acupuncture needles were inserted. Baihui (GV20) was punctured horizontally at a depth of 12–15 mm. Sishencong (EX-HN1) was punctured obliquely at a 15° angle toward Baihui (GV20), at a depth of 12–15 mm. Shenting (GV24) was punctured obliquely, at a depth of 8–10 mm. Sanyinjiao (SP6) was punctured perpendicularly at a depth of 15–20 mm. Taichong (LR3) was punctured perpendicularly at a depth of 12–15 mm. Manual stimulation with uniform lifting, thrusting, twisting, and flat tonifying techniques was applied until the patient experienced sensations of soreness, heaviness, or distension (sense of “deqi”).
3. Treatment time and frequency: Each session lasted 30 minutes. Treatments were administered every other day, three times per week, for a total of 4 weeks. Follow-up assessment was performed at 8 weeks after completion of treatment.

##### 2.2.4.3 AC group

1. The acupoint location, selection, and manipulation in this group are the same as those of the Acu group. Wet cupping acupoint selection referring to clinical experience (29), included Xinshu (BL15, bilateral) and Geshu (BL17, bilateral). Both acupoints could regulate qi and blood to address the core TCM pathogenesis of emotional disorders involving qi-blood imbalance. Xinshu (BL15) could calm the mind and stabilize mental activity to alleviate depressive manifestations like low mood and restlessness (30). Geshu (BL17)could nourish and harmonize blood to relieve stagnation-related emotional distress (31).
2. Following acupuncture intervention, wet cupping therapy was administered to patients in a prone posture by the same acupuncturists who performed the acupuncture procedure. First, the bilateral acupoints of Xinshu (BL15, bilateral) and Geshu (BL17, bilateral) were sanitized three consecutive times with cotton swabs saturated in 75% ethanol; next, a single-headed disposable plum blossom needle (Hwato, produced by Suzhou Medical Supply Factory Co., Ltd., Suzhou, China; Medical Device Registration No.: Su Xie Zhu Zhun 20162200970) was utilized to tap the disinfected acupoints 100–120 times with a force of 0.5 N until minimal bleeding (1–2 drops) emerged at the sites. Subsequently, 50 mL glass cups with a negative pressure of -0.04 MPa were applied to the tapped acupoints for blood collection and retained for 10 minutes, after which pre-weighed filter paper (Whatman No. 1) was employed to absorb the exuded blood and re-weighed post-treatment, with the blood volume determined by the conversion principle that 1 mg of weight increment is equivalent to 1 μL of blood. The target blood collection volume per acupoint was set at 1–2 mL, and the negative pressure of the cups was adjusted downward if the collected volume exceeded 3 mL. Finally, sterile gauze was applied to the cupping sites to prevent infection, and patients were instructed to avoid bathing within 24 hours after the treatment (32).
3. Treatment frequency: Wet cupping was performed every 3 days, twice per week, for 4 consecutive weeks. Follow-up assessment was conducted at 8 weeks after completion of treatment.

#### 2.2.5 Outcomes

##### 2.2.5.1 Primary outcome

The primary outcome was the patients’ change from baseline (pre-treatment) in the HAMD-24 (33) score at the end of week 4 (post-treatment). The HAMD-24 consists of 24 items across seven dimensions: anxiety/somatization, circadian rhythm disturbance, psychomotor retardation, sleep disorder, negative cognition, body image perception, and cognitive function. Scores are interpreted as follows: normal: ≤ 8 points, mild depression: 9–20 points, moderate depression: 21–35 points, severe depression: > 35 points.

##### 2.2.5.2 Secondary outcomes

Secondary outcomes included depression scales measured by the Self-Rating Depression Scale (SDS, 20 items; total score categorized as: normal ≤52, mild: 53-62, moderate: 63-72, severe: ≥73) (34), and the Montgomery-Asberg Depression Rating Scale (MADRS, 10 items; total score 0-60, higher scores indicates more severe depression) (35); neurological deficit was assessed using the National Institute of Health Stroke Scale (NIHSS, 11 items; total score 0-42, categorized as mild: 1-4, moderate: 5-10, moderate to severe: 16-20, severe: >20) (36). The aforementioned secondary outcomes were administered at baseline (pre-treatment), the end of week 4 (post-treatment), and 8 weeks after the 4-week treatment period (follow-up). Additionally, the change from baseline in the HAMD-24 score at the follow-up was defined as a secondary outcome.

##### 2.2.5.3 Hematological indicator outcomes

Tested by the Laboratory Department of Shanghai Shuguang Hospital Affiliated to Shanghai University of Traditional Chinese Medicine, the items include: Cort, ACTH, Interleukin (IL) (including IL-1β, IL-2, IL-6, IL-8, IL-10, IL-17), Tumor necrosis factor-α (TNF-α). Hematological indicators were assessed at baseline (pre-treatment) and at the end of week 4 (post-treatment).

#### 2.2.6 Safety assessment

Adverse events (e.g., local bleeding, dizziness) were monitored at each visit during the 4-week treatment period, categorized as treatment-related or unrelated.

#### 2.2.7 Randomization

A simple randomization method was used with SPSS 29.0 software (SPSS Inc., Chicago, IL, USA). To prevent selection bias, the generation of the randomization sequence was performed by an independent statistician who had no involvement in participant recruitment, while the assignment of participants was carried out by a separate researcher responsible for enrollment. Allocation concealment was ensured through the use of sequentially numbered, opaque, sealed envelopes. Each envelope contained a card indicating the group assignment (Acu or AC) and was opened only after eligibility confirmation and informed consent had been obtained. Participants were assigned to either the Acu group or the AC group in the order of their visit. To reduce subjective bias, blinding control was implemented for assessors and statisticians. At the same time, information isolation was ensured among acupuncturists, case collection physicians, and data statisticians.

#### 2.2.8 Blinding

Given the study design, participants and acupuncturists were not blinded to group assignments. To avoid measurement bias, the following procedures were strictly implemented:

1. Blinding of outcome assessors: All outcome assessments, including administration of HAMD-24, SDS, MADRS, NIHSS scales and collection of blood samples for hematological indicators, were performed by trained assessors who were unaware of the participants’ group allocation. Assessors had no involvement in any treatment procedures and were prohibited from communicating with acupuncturists or participants about group assignments during the trial period.
2. Standardized training for assessors and interventions: Prior to the trial initiation, all assessors and acupuncturists completed unified training program covering the standardized administration of rating scales, operational procedures for blood sample collection, acupuncture and wet cupping manipulation, and blinding protocols.
3. Centralized data management: Collected data were entered into database by a dedicated data manager who was also blinded to group assignments. Data entry was verified through double-entry to minimize errors, and any discrepancies were resolved by cross-referencing with original records without accessing group allocation information.
4. Standardized laboratory testing: Hematological indicators were analyzed by the Laboratory Department of Shanghai Shuguang Hospital Affiliated to Shanghai University of Traditional Chinese Medicine. Laboratory technicians were unaware of the participants’ group identities, and samples were labeled with unique codes rather than group information to ensure objective testing. At the same time, information isolation was ensured among acupuncturists, case collection physicians, and data statisticians.

#### 2.2.9 Statistical analysis

The minimum sample size was estimated using PASS 15.0 software (Number Cruncher Statistical Systems LLC, Kaysville, UT, USA). This sample size estimation was informed by a comprehensive literature review and results from preliminary pilot experiments. Based on the primary outcome (the change from baseline in the HAMD-24 score at the end of week 4), a total of 130 participants was estimated to provide 80% statistical power at a 2-sided significance level of 5% to detect a between-group difference of 8.8 points in the HAMD-24 score, assuming a standard deviation of 5.0 and a 20% dropout rate (37). This study used SPSS 29.0 (SPSS Inc., Chicago, IL, USA) for data processing and analysis. For quantitative data, it is presented in the form of mean ± standard deviation, and normality and homogeneity of variance tests are conducted first. If the data follows a normal distribution and the variance is uniform, independent sample t-test is used for inter group comparison; On the contrary, Mann Whitney U test is used. In the comparison of pre-treatment, post-treatment, and follow-up data within the group, paired t-test was used for normally distributed data, while Wilcoxon signed rank test was used for non-normally distributed data. Count data is expressed as percentages or composition ratios, comparison of intergroup rates is done using chi square tests, and rank sum tests are used for rank data. All statistical analyses were conducted at a significance level of P<0.05.

## 3 Results

### 3.1 Participant characteristics

**Figure 1:**
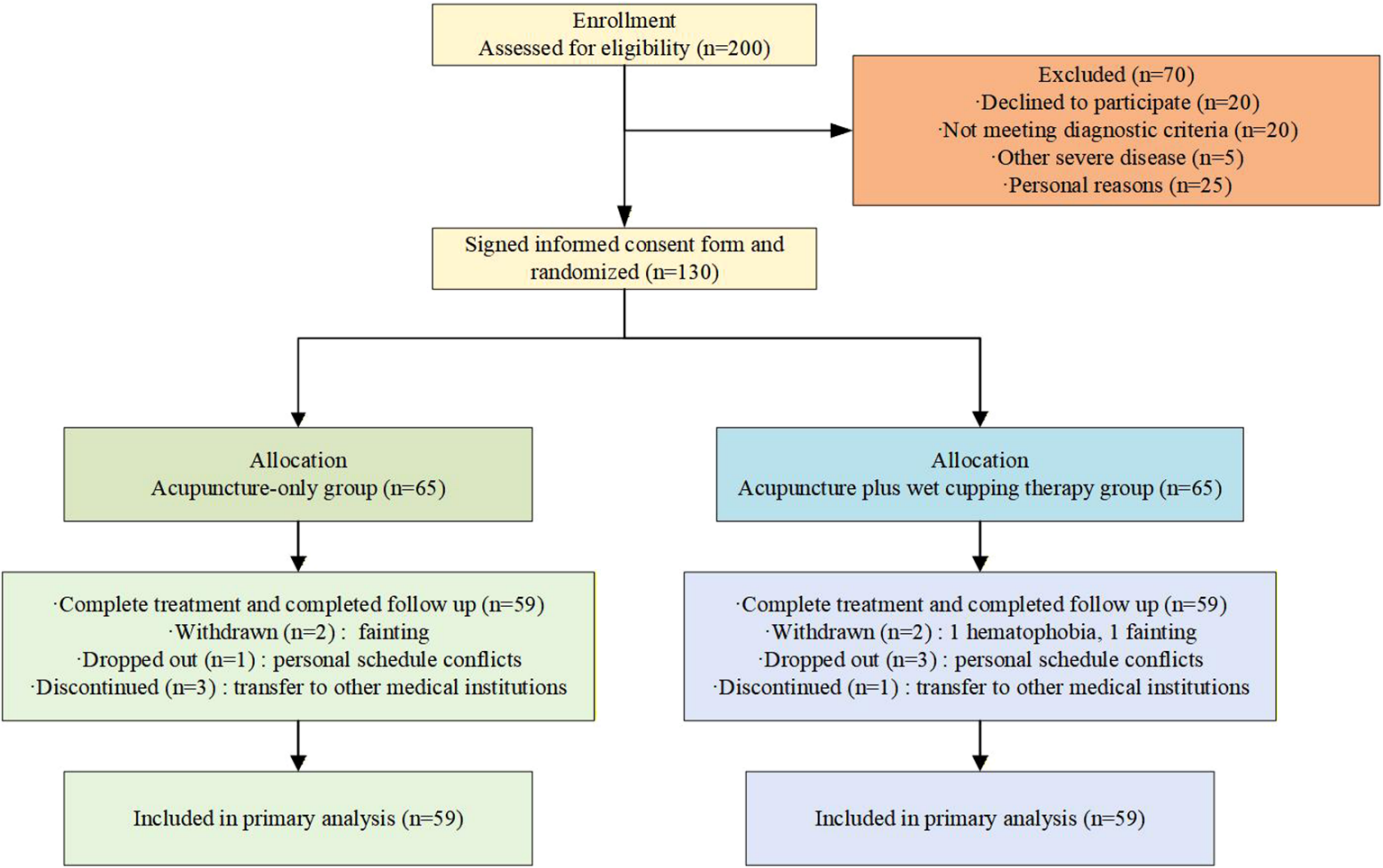
CONSORT Trial flow diagram.

The trial was conducted in strict adherence to the pre-approved protocol and CONSORT guidelines. A total of 200 participants were screened, of whom 70 were excluded based on the eligibility criteria, shown in **Figure 1**. The remaining 130 participants were enrolled and randomly assigned to the two groups. During the study, 12 participants did not complete the trial: 4 participants dropped out voluntarily (3 cases in the AC group and 1 case in the Acu group, due to personal schedule conflicts unrelated to treatment efficacy or safety), 4 participants were withdrawn (1 case of hematophobia in the AC group, 1 case of fainting in the AC group, 2 cases of fainting in the Acu group), and 4 participants discontinued treatment (1 in the AC group and 3 in the Acu group), owing to transfer to other medical institutions for subsequent management, which precluded their adherence to the scheduled treatment regimen. No protocol violations were identified among the enrolled participants. Ultimately, 118 participants completed the trial. **Table 1** presents the baseline features of the two groups. No significant differences were identified in demographic or clinical characteristics between the two groups (P>0.05).

**Table 1:** Demographic characteristics of the participants.

Variable	AC group (n=59)	Acu group (n=59)	P-value
Gender			0.71
Male/no. (%)	37 (62.71)	35 (59.32)	
Female/no. (%)	22 (37.29)	24 (40.68)	
Age/years [Mean (SD)]	65.34 (9.96)	63.93 (11.21)	0.47
Stroke classification/No. (%)			1.00
Cerebral hemorrhage	8 (13.56)	8 (13.56)	
Cerebral infarction	51 (86.44)	51 (86.44)	
Depression level/No. (%)			0.36
Mild	29 (49.15)	24 (40.68)	
Moderate	30 (50.85)	35 (59.32)	
Baseline			
HAMD-24 Scores, [Mean (SD)]	21.02 (3.66)	21.81 (4.47)	0.29
SDS Scores [M (P25, P75)]	58.75 (56.25, 61.88)	60.00 (56.25, 66.25)	0.31
NIHSS Scores [M (P25, P75)]	8.00 (7.00, 9.00)	8.00 (6.50, 9.00)	0.33
MADRS Scores [M (P25, P75)]	20.00 (18.00, 24.00)	20.00 (20.00, 22.00)	0.72
Cort (nmol/L), [Mean (SD)]	471.78 (133.41)	483.03 (117.38)	0.63
IL-1β (pg/ml), [Mean (SD)]	25.81 (9.05)	26.07 (9.22)	0.88
ACTH (pg/ml), [M (P25, P75)]	30.44 (18.95, 45.42)	29.53 (19.69, 45.08)	0.92
IL-2 (pg/ml), [M (P25, P75)]	1.60 (1.40, 1.96)	1.62 (1.33, 1.83)	0.28
IL-6 (pg/ml), [M (P25, P75)]	3.61 (2.69, 5.77)	3.24 (1.84, 5.46)	0.17
IL-8 (pg/ml), [M (P25, P75)]	12.47 (6.33, 20.30)	12.92 (6.32, 18.31)	0.90
IL-10 (pg/ml), [M (P25, P75)]	1.10 (0.91, 1.33)	1.08 (1.01, 1.25)	0.59
IL-17 (pg/ml), [M (P25, P75)]	3.87 (3.04, 5.38)	4.14 (3.12, 5.46)	0.75
TNF-α (pg/ml), [M (P25, P75)]	3.02 (2.06, 3.58)	2.89 (2.49, 3.62)	0.40

Acu, acupuncture; AC, acupuncture plus wet cupping; ACTH, adrenocorticotropic hormone; Cort, cortisol; HAMD-24, Hamilton Depression Scale-24 items; IL, interleukin; M, median; MADRS, Montgomery-Asberg Depression Rating Scale; NIHSS, National Institute of Health Stroke Scale; P25, 25th percentile; P75, 75th percentile; SD, standard deviation; SDS, Self-rating Depression Scale.

The primary analysis was conducted on the per-protocol (PP) population, which comprised only participants who completed the full course of treatment and all scheduled follow-up assessments (n=118), with exclusion of those who discontinued treatment or failed to adhere to the study protocol. No imputation was performed for missing data given the strict inclusion criteria of the PP population.

### 3.2 Outcomes

#### 3.2.1 Primary outcome

For primary outcome, mean HAMD-24 scores, which at baseline were 21.02 (SD, 3.66) in the AC group and 21.81 (SD, 4.47) in the Acu group, decreased to 12.03 (SD, 3.85) and 15.83 (SD, 3.17), respectively, at post-treatment, with a difference in change in HAMD-24 of -3.00 (95%CI: -4.14 to -1.86; P<0.01), as shown in **Table 2**.

**Table 2:** Changes in primary and secondary outcomes between AC group and Acu group.

Outcomes	AC group (n=59)	Acu group (n=59)	Difference (95% CI)	P-value
Change in HAMD-24 Scores from baseline, [Mean (SD)]				
Post-treatment	-8.99 (3.64)	-5.98 (2.54)	-3.00 (-4.14 to -1.86)	<0.01
Follow-up	-9.78 (4.11)	-6.38 (2.83)	-3.39 (-4.68 to -2.10)	<0.01
Change in SDS Scores from baseline, [M (P25, P75)]				
Post-treatment	-21.25 (-23.75, -16.25)	-10.00 (-14.38, -6.88)	-8.75 (-11.25 to -7.50)	<0.01
Follow-up	-22.50 (-25.00, -18.12)	-11.25 (-14.38, -6.25)	-11.25 (-12.50 to -8.75)	<0.01
Change in NIHSS Scores from baseline, [M (P25, P75)]				
Post-treatment	-3.00 (-4.00, -3.00)	-2.00 (-2.00, -1.00)	-2.00 (-2.00 to -1.00)	<0.01
Follow-up	-5.00 (-5.00, -4.00)	-3.00 (-3.50, -2.00)	-2.00 (-2.00 to -1.00)	<0.01
Change in MADRS Scores from baseline, [M (P25, P75)]				
Post-treatment	-11.00 (-13.00, -10.00)	-6.00 (-7.00, -5.00)	-6.00 (-6.00 to -5.00)	<0.01
Follow-up	-13.00 (-15.00, -11.50)	-8.00 (-9.00, -7.00)	-5.00 (-6.00 to -5.00)	<0.01

Acu, acupuncture; AC, acupuncture plus wet cupping; HAMD-24, Hamilton Depression Scale-24 items; M, median; MADRS, Montgomery-Asberg Depression Rating Scale; NIHSS, National Institute of Health Stroke Scale; P25, 25th percentile; P75, 75th percentile; SD, standard deviation; SDS, Self-rating Depression Scale.

#### 3.2.2 Secondary outcomes

For secondary outcomes, the follow-up HAMD-24 scores decreased to 11.24 (SD, 3.67) in the AC group and 15.42 (SD, 3.49) in the Acu group, with a between-group difference in HAMD-24 change of -3.39 (95%CI: -4.68 to -2.10; P<0.01).

SDS scores, which at baseline showed no between-group difference, decreased to 40.00 (33.75, 42.50) in the AC group and 48.75 (47.50, 51.25) in the Acu group at post-treatment, with a difference in change of -8.75 (95%CI: -11.25 to -7.50; P<0.01). Over follow-up, the difference in change from baseline in SDS between the two groups widened to -11.25 (95%CI: -12.50 to -8.75; P<0.01), with scores further decreasing to 37.50 (35.00, 41.25) in the AC group and increasing to 50.00 (47.50, 52.50) in the Acu group.

NIHSS scores, which at baseline showed no between-group difference, decreased to 5.00 (4.00, 5.50) in the AC group and 6.00 (5.00, 7.00) in the Acu group at post-treatment, with a difference in change of -2.00 (95%CI: -2.00 to -1.00; P<0.01). Over follow-up, the difference in change from baseline in NIHSS between the AC and Acu groups remained -2.00 (95%CI: -2.00 to -1.00; P<0.01), with scores changing to 4.00 (3.00, 4.00) in the AC group and 5.00 (4.00, 6.00) in the Acu group.

MADRS scores, which at baseline showed no between-group difference, decreased to 9.00 (7.50, 12.00) in the AC group and 14.00 (13.50, 16.00) in the Acu group at post-treatment, with a difference in change of -6.00 (95%CI: -6.00 to -5.00; P<0.01). Over follow-up, the difference in change from baseline in MADRS between the two groups changed to -5.00 (95%CI: -6.00 to -5.00; P<0.01), with scores changing to 7.00 (6.00, 9.00) in the AC group and 12.00 (12.00, 13.00) in the Acu group, as shown in **Table 2**.

#### 3.2.3 Hematological indicator outcomes

For biochemical and cytokine indicators, Cort, ACTH, IL-1β, IL-2, IL-6, IL-8, IL-10, IL-17, and TNF-α levels showed no significant between-group differences at baseline (all P>0.05). Both groups exhibited significant post-treatment changes in these indicators, with more notable adjustments observed in the AC group.

Cort levels, which were 471.78 ± 133.41 nmol/L in the AC group at baseline, decreased to 362.67 ± 118.32 nmol/L at post-treatment, with a between-group difference in change of -60.94 nmol/L (95%CI: -76.42 to -45.47; P<0.01) compared with the Acu group. ACTH levels, which showed no between-group difference at baseline, changed to 16.32 (11.65, 26.44) in the AC group at post-treatment, with a median between-group difference in change of -4.12 (95%CI: -6.95 to -1.27; P<0.01) compared with the Acu group [25.64 (15.88, 34.72)].

For pro-inflammatory cytokines, the AC group had a greater reduction than the Acu group at post-treatment, with the following between-group differences in change: IL-1β levels: mean difference in reduction of -3.96 pg/ml (95%CI: -5.38 to -2.55; P<0.01); IL-2 levels: median difference in reduction of -0.23 pg/ml (95%CI: -0.31 to -0.13; P<0.01); IL-6 levels: median difference in reduction of -1.29 pg/ml (95%CI: -1.68 to -0.88; P<0.01); IL-8 levels: median difference in reduction of -2.60 pg/ml (95%CI: -4.45 to -1.02; P<0.01); IL-17 levels: median difference in reduction of -0.68 pg/ml (95%CI: -1.09 to -0.36; P<0.01); TNF-α levels: median difference in reduction of -0.32 pg/ml (95%CI: -0.64 to -0.07; P<0.01). For the anti-inflammatory cytokine, IL-10 levels showed a larger increase in the AC group than in the Acu group at post-treatment, with a median between-group difference in change of 0.20 pg/ml (95%CI: 0.10 to 0.31; P<0.01), as shown in **Table 3**.

**Table 3:** Changes in hematological indicators between AC group and Acu group.

Outcomes	AC group (n=59)	Acu group (n=59)	Difference (95% CI)	P-value
Change in Cort from baseline to post-treatment (nmol/L), [Mean (SD)]	-109.10 (52.22)	-48.16 (29.26)	-60.94 (-76.42 to -45.47)	<0.01
Change in IL-1β from baseline to post-treatment (pg/ml), [Mean (SD)]	-7.38 (5.06)	-3.42 (2.05)	-3.96 (-5.38 to -2.55)	<0.01
Change in ACTH from baseline to post-treatment (pg/ml), [M (P25, P75)]	-9.74 (-17.58, -4.89)	-5.16 (-10.04, -2.72)	-4.12 (-6.95 to -1.27)	<0.01
Change in IL-2 from baseline to post-treatment (pg/ml), [M (P25, P75)]	-0.35 (-0.63, -0.22)	-0.25 (-0.38, -0.01)	-0.23 (-0.31 to -0.13)	<0.01
Change in IL-6 from baseline to post-treatment (pg/ml), [M (P25, P75)]	-1.93 (-3.23, -1.46)	-0.70 (-1.66, -0.21)	-1.29 (-1.68 to -0.88)	<0.01
Change in IL-8 from baseline to post-treatment (pg/ml), [M (P25, P75)]	-6.46 (-10.21, -2.25)	-2.79 (-6.83, -0.76)	-2.60 (-4.45 to -1.02)	<0.01
Change in IL-10 from baseline to post-treatment (pg/ml), [M (P25, P75)]	0.45 (0.19, 0.67)	0.21 (0.11, 0.34)	0.20 (0.10 to 0.31)	<0.01
Change in IL-17 from baseline to post-treatment (pg/ml), [M (P25, P75)]	-1.26 (-2.52, -0.65)	-0.67 (-0.89, -0.36)	-0.68 (-1.09 to -0.36)	<0.01
Change in TNF-α from baseline to post-treatment (pg/ml), [M (P25, P75)]	-0.79 (-1.71, -0.41)	-0.60 (-0.85, -0.28)	-0.32 (-0.64 to -0.07)	<0.01

Acu, acupuncture; AC, acupuncture and wet cupping; ACTH, adrenocorticotropic hormone; Cort, cortisol; IL, interleukin; M, median; P25, 25th percentile; P75, 75th percentile; SD, standard deviation.

### 3.3 Adverse events

During the trial, 4 adverse reactions occurred in the AC group (1 case of hematophobia, 1 case of fainting during treatment, 1 case of subcutaneous hematoma, and 1 case of subcutaneous pain), and 4 adverse reactions occurred in the Acu group (2 cases of fainting during treatment and 2 cases of subcutaneous hematoma). These adverse events were classified as mild to moderate and were not considered protocol violations, as they were consistent with the known safety profile of acupuncture and wet cupping therapies; furthermore, relevant information had been disclosed to participants prior to enrollment, and written informed consent was obtained. Among them, 4 patients with subcutaneous hematoma and subcutaneous pain in the two groups did not experience adverse reactions after timely symptomatic treatment (local compression for hematoma, ice compress for pain), and the patients were willing to continue the treatment. A total of 4 patients with hematophobia and fainting during treatment in the two groups indicated that they could not continue to cooperate with the treatment and were withdrawn. No serious adverse events occurred during the entire trial, and no protocol deviations related to treatment administration, outcome assessment, or data collection were observed.

## 4 Discussion

### 4.1 Comparative efficacy of combined therapy versus acupuncture monotherapy

The therapeutic efficacy of Traditional Chinese Medicine (TCM) in alleviating depressive symptoms, particularly in PSD, is supported by both clinical and biological evidence (38, 39). Both interventions alleviated depressive symptoms and improved neurological function, but the AC therapy exerted more robust and durable effects, which may be attributed to synergistic regulation of the HPA axis and inflammatory responses. Notably, while SDS scores in the Acu group rebounded slightly during follow-up, both groups maintained significant improvements (P<0.01). Effect sizes exceed those reported for SSRIs in mild PSD (Cohen’s d ~0.6-0.8) while showing superior safety (40, 41). These findings underscore the synergistic potential of combining TCM therapies with conventional treatments for PSD.

In addition to depressive symptoms, neurological recovery was also enhanced. NIHSS scores declined more significantly in the AC group than in the Acu group during treatment (P<0.01), and this trend persisted at follow-up (P<0.01). These findings suggest that integrative TCM therapies may not only target emotional disturbances but also promote neurological rehabilitation, possibly through multimodal mechanisms involving neuroendocrine and inflammatory regulation.

### 4.2 Synergistic mechanisms of wet cupping therapy

Wet cupping therapy exerted distinct therapeutic effects in PSD by targeting both depressive symptoms and the underlying pathological processes. First, compared to acupuncture alone, the combination therapy produced more pronounced reductions in depression-specific scales, suggesting that wet cupping directly enhances the alleviation of core depressive manifestations. Second, the significant improvements in neurological function (NIHSS) implied that wet cupping may synergize with acupuncture to promote neural recovery after stroke, which in turn mitigates depressive symptoms through breaking the “stroke-disability-depression” vicious cycle. Third, the more substantial regulation of HPA-axis hyperactivity and inflammatory dysregulation confirmed that wet cupping addresses the biological underpinnings of PSD, beyond mere symptomatic relief. These effects are consistent with clinical observations that wet cupping serves as an effective complementary therapy for mood disorders, particularly in conditions associated with neuroinflammation and neuroendocrine imbalance.

### 4.3 Potential mechanisms underlying the efficacy of wet cupping therapy

The synergistic effects of wet cupping may be attributed to multiple interrelated biological mechanisms, primarily involving the regulation of the neuroendocrine-immune network, improvement of local microcirculation, and modulation of inflammatory signaling pathways:

Regulation of the HPA axis and neuroendocrine balance: Wet cupping’s negative pressure and mild skin stimulation activate peripheral nerve endings, which transmit signals to the central nervous system to inhibit excessive activation of the HPA axis (42). Cupping therapy reduces cerebrospinal fluid cortisol levels (42), which aligns with our findings of greater reductions in Cort and ACTH in the AC group. This regulation may be mediated by enhanced release of endogenous opioids and modulation of central neurotransmitter systems, thereby restoring the balance between the HPA axis and the sympathetic-adrenal-medullary system (43).

Improvement of microcirculation and resolution of “blood stasis”: From the perspective of traditional Chinese medicine, PSD is often associated with “qi stagnation and blood stasis” (8). Wet cupping promotes local blood circulation through negative pressure suction, accelerates the clearance of metabolic waste and inflammatory mediators, and improves tissue oxygenation and nutrient supply (42). This mechanical stimulation enhances microvascular perfusion, reduces leukocyte aggregation, and alleviates tissue edema (42), which not only relieves local discomfort but also facilitates neural tissue repair and functional recovery. The improvement of systemic microcirculation further contributes to the regulation of the neuroendocrine-immune network, forming a synergistic therapeutic effect.

### 4.4 Synergistic mechanisms between wet cupping and acupuncture

Acupuncture primarily regulates central neurotransmitter systems and HPA-axis function, while wet cupping focuses on peripheral immune modulation and microcirculation improvement. Their combination achieves a “central-peripheral dual regulation” effect: acupuncture targets the central nervous system to normalize neuroendocrine function, and wet cupping modulates peripheral inflammatory responses and microcirculation to eliminate pathological stimuli. This dual-targeted approach addresses the multi-pathway pathogenesis of PSD (neuroendocrine dysregulation, neuroinflammation, and microcirculation disturbance), leading to superior therapeutic effects compared to single therapy. Additionally, the mechanical stimulation of wet cupping may enhance the sensitivity of acupoints to acupuncture, promoting the conduction of “qi” and strengthening the regulatory effects on the body’s homeostasis (44).

### 4.5 Role of HPA axis dysfunction in PSD and regulatory effects of combined therapy

Dysfunction of the HPA axis is widely considered a key mechanism underlying PSD pathogenesis and is a hallmark of severe depression (45). Hypersecretion of Cort and ACTH reflects HPA axis hyperactivity, which occurs in nearly half of patients with depression (46). When organisms encounter physiological or psychological stressors, the HPA axis exhibits a characteristic cascade activation pattern: neurons in the paraventricular nucleus of the hypothalamus secrete corticotropin releasing hormone (CRH), which passes through the pituitary portal system and first reaches the pituitary gland, promoting ACTH production; Subsequently, ACTH enters the bloodstream and acts on the corticospinal fasciculus cells, promoting the terminal synthesis and release of Cort. This multi-level neuroendocrine regulation not only forms a complete stress feedback loop, but also synergistically activates the sympathetic nervous system, triggering a series of physiological responses (47). After stroke, the HPA axis exhibits an abnormally hyperactive state, which leads to a large secretion of Cort. Cort can promote the synthesis of tryptophan pyrrolidine and transaminase in the liver. In this process, Cort also accelerates the catabolism of 5-HT precursor tryptophan and NE precursor tyrosine, ultimately leading to a decrease in monoamine neurotransmitter levels. Monoamine neurotransmitters play a crucial role in neural transmission, and a decrease in their concentration can interfere with the neural transmission process in the hippocampus. This disruption of neural transmission may ultimately lead to the induction of depressive symptoms (48, 49). This reaction will also terminate correspondingly after the pressure source disappears. In this cycle, Cort levels decrease and inhibit further release of ACTH and CRH, playing a critical role in regulating emotions in the hippocampus (50). When patients with depression are in the onset stage, their HPA axis function is significantly overactive, and when they are in the remission stage, HPA axis function improves significantly.

Both therapies reduced Cort and ACTH levels (P<0.01), with the AC group achieving more robust suppression, suggesting superior HPA axis normalization. The levels of Cort and ACTH in the serum of PSD patients may be positively correlated with the severity of their depressive state. Compared with healthy individuals, the serum Cort and ACTH concentrations in depressed patients are at higher levels (11, 51), and after antidepressant treatment, Cort and ACTH levels generally decrease (52). It can be considered that patients with depression exhibit more persistent HPA axis overactivity, leading to elevated levels of Cort and ACTH. Simply put, HPA axis disorders are associated with the risk of depression. By detecting the levels of Cort and ACTH in the blood, the HPA axis function of the body can be reflected to some extent, which can be used to assist in determining the severity of PSD and evaluate the effectiveness of treatment.

### 4.6 Pathological role of inflammatory cytokines in PSD and regulatory effects of combined therapy

HPA axis dysfunction is not the only cause of PSD, as high oxidative stress, immune response, and neuroinflammation are also associated with depression (13). Pro-inflammatory cytokines, particularly IL-1β, IL-2, IL-6, IL-8, IL-17, and TNF-α, play pivotal roles in PSD pathogenesis. Their elevated levels correlate with oxidative stress, neuronal apoptosis, and disrupted synaptic plasticity. After stroke, the intestinal barrier is damaged, and the resulting translocation of lipopolysaccharides (LPS) activates immune cells, which secrete various pro-inflammatory cytokines. This process can disrupt the normal functioning of neurotransmitters, affect synaptic activity of neurons, and increase the risk of developing depression (18–20). Post treatment, both interventions significantly reduced these cytokines, with the AC group showing greater efficacy (P< 0.01). In contrast, the anti-inflammatory cytokine IL-10 exhibited a distinct pattern, increasing significantly after therapy (P< 0.01), particularly in the AC group (53, 54). A study by Chi et al. (54) found that IL-10 levels in PSD patients were significantly reduced, and proposed that the optimal critical value of IL-10 is 0.615pg/mL, which can be considered as a reference indicator for PSD. The level of IL-10 is also associated with the severity of stroke and the degree of recovery after stroke. This dichotomy underscores the importance of differentiating pro- and anti-inflammatory factors in evaluating PSD interventions.

### 4.7 Crosstalk between HPA axis and inflammatory responses in PSD and intervention value of combined therapy

The crosstalk between HPA axis dysfunction and immune inflammation forms a vicious cycle in PSD. HPA overactivation amplifies pro-inflammatory cytokine release, which in turn inhibits glucocorticoid receptor (GR) function, further dysregulating the HPA axis (45, 55, 56). Conversely, inflammatory cytokines impair hippocampal neurogenesis, exacerbating depression (57, 58). The AC intervention’s dual suppression of Cort/ACTH and pro-inflammatory cytokines highlights its capacity to disrupt this cycle, while IL-10 upregulation may promote anti-inflammatory feedback (53). These findings collectively support a paradigm wherein combined therapies target both neuroendocrine and immune pathways, offering a comprehensive approach to PSD management. Notably, these results demonstrate the comparative advantage of the combined therapy over acupuncture monotherapy, and do not rule out the potential contribution of non-specific effects (e.g., expectation, therapist attention, contextual factors) to the observed therapeutic benefits.

### 4.8 Limitation

A couple of limitations of this study should be noted. First, the study only compared acupuncture alone with acupuncture plus wet cupping, and did not include a sham control, placebo control, or usual-care-only group. Thus, the trial only demonstrates incremental benefit over acupuncture monotherapy rather than absolute efficacy for PSD, and non-specific effects (e.g., patient expectation, therapist attention, contextual factors) cannot be excluded from the observed therapeutic outcomes. Second, the trial was conducted at a single center with a modest sample size (118 participants completed the study), which may restrict the broader applicability of the results. Differences in patient demographics and clinical practice across regions could also affect the outcomes. Third, although allocation concealment and formal blinded assessment were implemented, participants and acupuncturists were not blinded to group assignments, which may introduce performance bias, expectancy bias, and potential assessment bias. Besides, the mechanisms underlying the synergistic effect of acupuncture and wet cupping were inferred based on changes in peripheral biomarkers rather than direct central nervous system (CNS) measurements. Future studies could incorporate neuroimaging (e.g., functional MRI) or cerebrospinal fluid analyses to better elucidate the CNS-specific pathways involved in the antidepressant and neuroprotective effects of AC therapy.

Given the exploratory pilot nature of this study, sensitivity analysis was not conducted to assess the robustness of the primary and secondary outcome results. Additionally, the intention-to-treat (ITT) population is the conventional choice for the primary analysis in RCTs to minimize selection bias and reflect the real-world treatment effect. However, the PP population was adopted for the primary analysis in the present pilot RCT, in consideration of the small sample size and the primary research objective of evaluating the preliminary efficacy of the intervention in participants with good protocol adherence. Our future large-scale, multi-center randomized controlled trials with sufficient statistical power will incorporate sensitivity analysis to verify the stability of the findings.

### 4.9 Clinical implications

From a clinical perspective, this study provides valuable evidence for the integration of traditional Chinese medicine therapies into PSD management. For clinicians treating mild-to-moderate PSD patients, particularly those with poor tolerance to conventional pharmacotherapy or incomplete response to single acupuncture, the combination of acupuncture and wet cupping offers a safe and effective alternative. The synergistic regulation of HPA axis and inflammatory balance by this combined therapy addresses the multi-pathway pathogenesis of PSD, which is crucial for achieving durable symptomatic relief and promoting neurological recovery. Additionally, the standardized acupoint selection and operational procedures described in this study (e.g., specific wet cupping sites at Xinshu BL15 and Geshu BL17, controlled negative pressure and blood collection volume) provide practical operability for clinical application, facilitating its dissemination in acupuncture and rehabilitation settings. Given the low incidence of serious adverse events, this combined therapy also exhibits favorable safety profiles, which is essential for improving long-term treatment adherence in PSD patients, a population often burdened by comorbidities and functional impairments. Furthermore, the findings highlight the potential of Cort, ACTH, and IL-10 as candidate biomarkers for monitoring treatment response, enabling clinicians to individualize therapeutic strategies and optimize clinical outcomes. Collectively, these results support the clinical utility of acupuncture plus wet cupping as a promising complementary intervention for PSD, enriching the current therapeutic armamentarium and addressing the unmet clinical needs for well-tolerated and effective treatments.

## 5 Conclusion

In this randomized trial, acupuncture plus wet cupping demonstrated comparative superiority over acupuncture monotherapy in improving PSD symptoms and neurological deficits in mild-to-moderate PSD patients. The combined therapy was also accompanied by favorable changes in Cort, ACTH, IL, and TNF-α. These results reflect incremental therapeutic benefits of the combined intervention rather than definitive efficacy beyond placebo or standard care for PSD. These findings provide preliminary evidence supporting the potential clinical value of acupuncture plus wet cupping for PSD.

## Data Availability

The original contributions presented in the study are included in the article/**Supplementary Material**. Further inquiries can be directed to the corresponding authors.
